# Iron Causes Lipid Oxidation and Inhibits Proteasome Function in Multiple Myeloma Cells: A Proof of Concept for Novel Combination Therapies

**DOI:** 10.3390/cancers12040970

**Published:** 2020-04-14

**Authors:** Jessica Bordini, Federica Morisi, Fulvia Cerruti, Paolo Cascio, Clara Camaschella, Paolo Ghia, Alessandro Campanella

**Affiliations:** 1 Division of Experimental Oncology, IRCCS San Raffaele Scientific Institute, 20132 Milan, Italy; bordini.jessica@hsr.it (J.B.); ghia.paolo@hsr.it (P.G.); 2 Fondazione Centro San Raffaele, 20132 Milan, Italy; 3 Division of Genetics and Cell Biology, IRCCS San Raffaele Scientific Institute, 20132 Milan, Italy; Fede85.federica@gmail.com (F.M.); camaschella.clara@hsr.it (C.C.); 4 Department of Veterinary Sciences, University of Turin, 10095 Grugliasco, Italy; fulvia.cerruti@unito.it (F.C.); paolo.cascio@unito.it (P.C.); 5 School of Medicine, Vita-Salute San Raffaele University, 20132 Milan, Italy

**Keywords:** multiple myeloma, iron, ferroptosis, proteasome

## Abstract

Adaptation to import iron for proliferation makes cancer cells potentially sensitive to iron toxicity. Iron loading impairs multiple myeloma (MM) cell proliferation and increases the efficacy of the proteasome inhibitor bortezomib. Here, we defined the mechanisms of iron toxicity in MM.1S, U266, H929, and OPM-2 MM cell lines, and validated this strategy in preclinical studies using Vk*MYC mice as MM model. High-dose ferric ammonium citrate triggered cell death in all cell lines tested, increasing malondialdehyde levels, the by-product of lipid peroxidation and index of ferroptosis. In addition, iron exposure caused dose-dependent accumulation of polyubiquitinated proteins in highly iron-sensitive MM.1S and H929 cells, suggesting that proteasome workload contributes to iron sensitivity. Accordingly, high iron concentrations inhibited the proteasomal chymotrypsin-like activity of 26S particles and of MM cellular extracts in vitro. In all MM cells, bortezomib-iron combination induced persistent lipid damage, exacerbated bortezomib-induced polyubiquitinated proteins accumulation, and triggered cell death more efficiently than individual treatments. In Vk*MYC mice, addition of iron dextran or ferric carboxymaltose to the bortezomib-melphalan-prednisone (VMP) regimen increased the therapeutic response and prolonged remission without causing evident toxicity. We conclude that iron loading interferes both with redox and protein homeostasis, a property that can be exploited to design novel combination strategies including iron supplementation, to increase the efficacy of current MM therapies.

## 1. Introduction

Cancer cells rearrange iron trafficking proteins to promote iron uptake and retention, to favor proliferation [1,2]. Accordingly, iron chelation has been proposed as a strategy to impair tumor growth [3,4]. The opposite holds true, since excess iron is toxic, even for cancer cells, because it catalyzes oxidative stress. Cellular defensive machinery against iron excess relies on increasing translation of the safe-iron storage protein ferritin, promoting degradation of the iron importer transferrin receptor 1 (TFR1) to inhibit further iron uptake and enhancing ferroportin protein on cell surface to favor iron export [5,6]. Notwithstanding this coordinated response, exposure to iron excess increases the cytosolic iron pool, oxidative stress, and consequent cell damage [7].

We have recently demonstrated the efficacy of exploiting iron toxicity to affect cancer cells growth in models of multiple myeloma (MM), a disease in which malignant plasma cells accumulate in the bone marrow and secrete monoclonal immunoglobulin, generating bone disease, organ failure and anemia [8]. Iron exposure triggers reactive oxygen species (ROS) formation, promoting cell death, impairing MM cell proliferation in vitro and disease expansion in vivo [9]. We also found that the proteasome inhibitor bortezomib, a compound commonly used in the setting of MM patients, impairs the physiologic response to iron excess, i.e., TFR1 degradation and ferritin increase, abolishing the cellular defensive response against iron exposure and maximizing the toxicity of supplemented iron [10]. Accordingly, bortezomib-iron combination impairs MM cell viability and disease expansion more efficiently than individual agent treatments [9,10], suggesting that the addition of high-dose iron to bortezomib-based pharmacological schedules is worth being explored to increase their therapeutic efficacy.

Iron is involved in multiple cellular processes and dysregulation of its homeostasis potentially interferes with several pro-survival adaptive mechanisms that occur in MM cells, including the maintenance of protein homeostasis. Exploring the role of iron in the latter process is particularly promising, considering our previous findings on additive effect of iron toxicity and proteasome inhibition. Protein homeostasis is finely tuned in MM cells in order to support high production of immunoglobulins through the secretory pathway [11,12]. MM cells must efficiently degrade misfolded immunoglobulins and protein aggregates to protect themselves from proteotoxic stress [13]. Abnormal proteins degradation occurs in a ubiquitin-dependent manner by the proteasome system, making the proteasome vulnerable in MM cells [14] and providing the rationale for the efficacy of proteasome inhibitors, such as bortezomib and carfilzomib, drugs that have significantly improved MM patient outcome. In this context, low proteasome capacity and high proteasome workload identify proteasome-inhibitor sensitive cells [15]. Autophagy contributes to proteostasis in MM: high basal levels of autophagy are essential for MM cell survival [16] and provide an alternative proteolytic pathway in response to proteotoxic stress induced by proteasome inhibition. Accordingly, accumulation of misfolded proteins by proteasome inhibition stimulates autophagy, an event that contributes to bortezomib resistance in some MM cells, such as U266 cells [17].

Based on our findings that iron excess negatively affects MM cell growth and that combination of iron with bortezomib increases treatment efficacy, here we investigated the mechanisms of iron toxicity. We studied the effect of iron excess on redox and protein homeostasis in MM cell lines whose iron sensitivity was previously validated, as the MM.1S and U266 cells [9], and in two additional cell lines, the H929 and OPM-2 cells, never tested before. Then, we proved the translational relevance of our findings by testing the effect of iron on carfilzomib efficacy and by adding high-dose iron to the common bortezomib-melphalan-prednisone (VMP) regimen in a preclinical study using the Vk*MYC mice as MM model [18].

## 2. Results

### 2.1. Iron Causes Lipid Damage and Impairs Proteasome Functionality in MM Cell Lines

**Figure 1:**
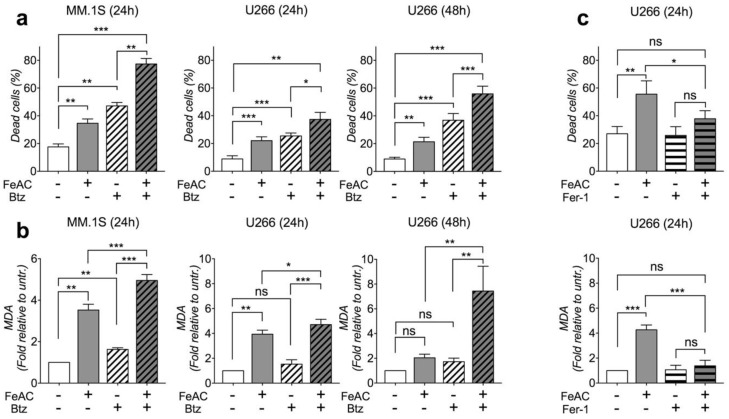
Iron triggers cell death by inducing lipid damage. (**a**,**b**) MM.1S and U266 cell lines were subjected to 600 μM ferric ammonium citrate (FeAC) or 10 nM bortezomib (Btz) or combination for 24 or 48 h. (**a**) Percentage of dead cells. (**b**) Malondialdehyde (MDA) levels presented as fold change relative to untreated cells. (**c**) Percentage of cell death and MDA levels in U266 cells treated or not with 100 μM ferrostatin (Fer-1) 2 h before 600 μM FeAC for 24 h. All samples were additionally supplemented with 0.1% DMSO. (**a**–**c**) Values are shown as mean ± standard errors of at least 4 independent experiments for each cell line. Statistical differences were determined by Tukey post-ANOVA test. ns: non-statistically significant. * *p* < 0.05; ** *p* < 0.01. *** *p* < 0.001.

We treated MM.1S, U266, H929 and OPM-2 cell lines with high iron concentration (600 µM ferric ammonium citrate, FeAC) or 10 nM bortezomib or FeAC plus bortezomib for 24 h. As an iron source, we utilized ferric iron (FeAC) that is commonly used for in vitro experiments with cells since it is stable at neutral pH and converted to bioactive ferrous iron inside the cells. In all cell lines, iron and bortezomib caused cell death, and their combination was more effective than treatment with single agents (Figure 1a and Figure S1A). U266 and OPM-2 appeared less sensitive to iron toxicity than MM.1S and H929. Treatment extension up to 48 h in the former cells did not exacerbate the effect of iron on cell viability, while it intensified bortezomib toxicity and the result of bortezomib-iron combination (Figure 1a and Figure S1A). In all cell lines analyzed at 24 h after treatment, iron strongly increased the levels of malondialdehyde (MDA), a by-product of lipid peroxidation and a recognized index of ferroptosis (Figure 1b and Figure S1B). In accordance with this observation, pre-treatment of U266 cells with 100 µM ferrostatin, a compound that prevents lipid peroxidation, abolished iron-induced MDA formation and iron toxicity (Figure 1c). Bortezomib had no evident effect on lipid peroxidation, while bortezomib-iron combination induced MDA formation above levels produced by iron alone in all cell lines (Figure 1b and Figure S1B). MDA formation was attenuated in U266 and OPM-2 cells treated with iron as a single agent for 48 h (Figure 1b and Figure S1B). Differently, lipid peroxidation was strongly enhanced upon bortezomib-iron combination (Figure 1b and Figure S1B). We concluded that iron exposure triggers cell death by inducing lipid damage and that this event is more evident and persistent after bortezomib treatment.

**Figure 2:**
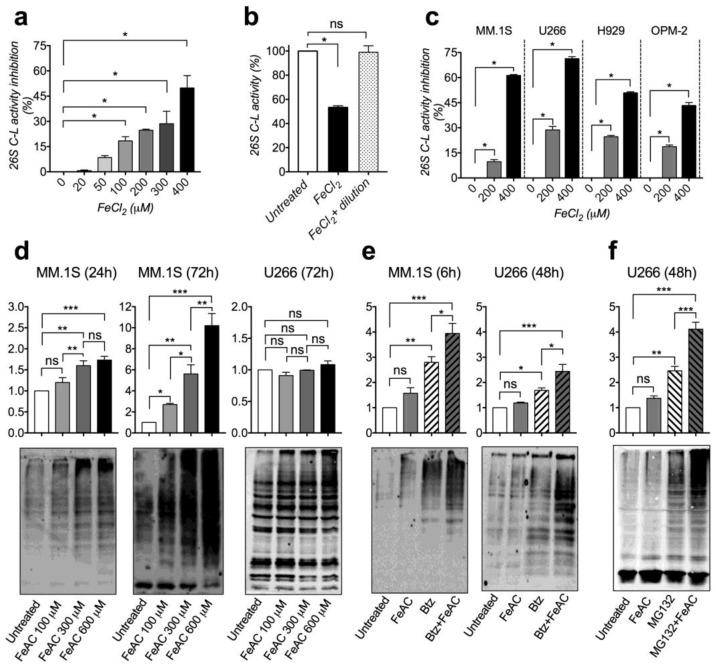
Iron impairs proteasomal activity and causes polyubiquitinated proteins accumulation. (**a**,**b**) Evaluation of chymotrypsin-like (C-L) activity of purified 26S proteasome after pre-incubation with titrated doses of ferrous chloride (FeCl_2_) for 5 min. (**a**) Data show the percentage of C-L activity inhibition. (**b**) Data show residual C-L activity after pre-incubation with 400 μM FeCl_2_ followed or not by iron dilution prior to C-L activity evaluation. (**c**) Evaluation of proteasomal C-L activity of multiple myeloma (MM) cellular extracts after pre-incubation with titrated doses of FeCl_2_ for 5 min. Background activity (caused by non-proteasomal degradation) was determined by addition of 2 µM epoxomicin and subtracted from total C-L activity. (**d**,**e**) Polyubiquitinated (Poly-Ub) proteins levels in: (**d**) MM.1S and U266 cells treated with titrated doses of ferric ammonium citrate (FeAC) for 24 or 72 h; (**e**) MM cells treated with 600 μM FeAC or 10 nM bortezomib (Btz) or combination for 6 h (MM.1S) or 48 h (U266); (**f**) U266 cells treated with 600 μM FeAC or 0.5 μM MG132 or combination for 48 h. Upper panels: summary of densitometry of at least 3 independent experiments (Fold relative to untreated). Lower panels: Representative western blotting. Values are shown as mean ± standard errors. (**a**–**c**) Statistical differences were determined by nonparametric Mann-Whitney U test. (**d**–**f**) Statistical differences were determined by Tukey post-ANOVA test. ns: non-statistically significant. * *p* < 0.05; ** *p* < 0.01. *** *p* <0.001.

Then, we investigated whether iron directly interferes with bortezomib activity by mechanistically exploring the effect of iron on proteasome activity. We carried out a biochemical study by using highly purified rabbit 26S proteasome that was pre-incubated with ferrous chloride or ferrous sulfate, at concentrations ranging from 20 μM to 400 μM, or with respective control anions. Ferrous iron recapitulates the bioactive iron-species that strongly increase within cells after iron exposure. Both ferrous iron formulations induced a dose-dependent inhibition of chymotrypsin-like activity, indicating that high iron concentration directly impairs proteasome functionality (Figure 2a and Figure S2A). The effect of iron was reversible since the dilution of iron after pre-incubation completely restored proteasome activity (Figure 2b and Figure S2B). Then, we evaluated the effect of iron on the whole chymotrypsin-like proteasomal activity of MM cell lines by pre-treating cellular extracts with 200 μM or 400 μM ferrous iron sources. In samples from all cell lines analyzed, both ferrous chloride and ferrous sulfate significantly inhibited proteasomal chymotrypsin-like activity in a dose-dependent manner (Figure 2c and Figure S2C). Therefore, we concluded that iron loading inhibits proteasome activity in MM cells.

To test whether proteasome impairment may occur in iron-exposed cells, we evaluated poly-ubiquitinated (poly-Ub) proteins levels in MM cell lines treated with titrated doses of FeAC (100, 300 and 600 μM) for 24 and 72 h. Iron caused poly-Ub protein accumulation in a dose-dependent manner in MM.1S and H929, the effect being detectable at 24 h and exacerbated by treatment extension (Figure 2d and Figure S2D). Poly-Ub accumulation was barely visible in U266 and OPM-2 cells (Figure 2d and Figure S2D). In parallel, we evaluated poly-Ub proteins levels in MM.1S and U266 cells treated with FeAC (600 μM) or bortezomib (10 nM) or combination, as described above. As expected, bortezomib caused poly-Ub proteins accumulation in both cell lines, confirming the proteasome impairment (Figure 2e). Addition of iron to bortezomib further increased poly-Ub proteins accumulation in both cell lines (Figure 2e). A similar result was obtained by treating U266 cells with FeAC (600 μM) and the proteasome inhibitor MG132 (0.5 μM) (Figure 2f). Altogether our results uncover that iron directly impairs proteasome functionality, an effect that adds to that of proteasome inhibitors.

**Figure 3:**
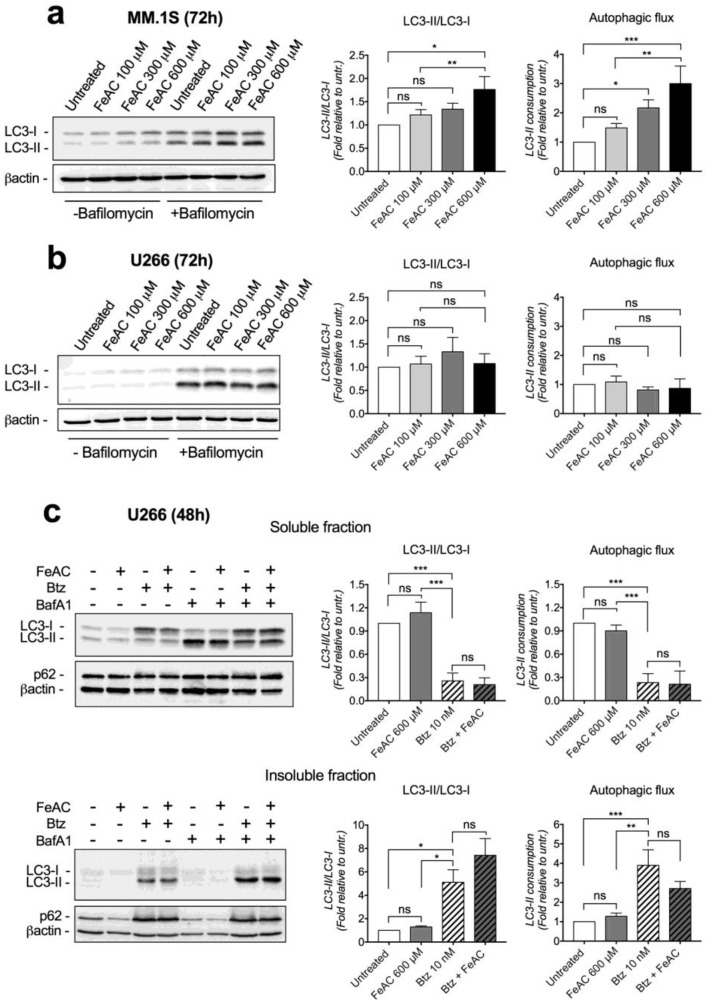
Long time iron exposure increases autophagy in MM.1S cells but does not increase autophagy response after proteasome inhibition. (**a**,**b**) LC3 protein levels measured in MM cell lines treated with titrated doses of ferric ammonium citrate (FeAC) for 72 h. (**c**) LC3 and p62 protein levels in soluble and insoluble protein extracts fractions obtained from U266 cells treated with 600 μM FeAC or 10 nM bortezomib (Btz) or combination for 48 h. Where indicated, bafilomycin (BafA1) was added at 75 nM for the last 8 h of incubation. The rate of autophagosome formation was estimated by considering LC3-II/LC3-I ratio in bafilomycin treated cells. Autophagic flux was considered as the difference of LC3-II protein between bafilomycin-treated and untreated cells. Left panels: representative western blots. Right panels: densitometry summaries of at least 3 independent replicates for each analysis. Values are shown as mean ± standard errors. Statistical differences were determined by Tukey post-ANOVA test. ns: non-statistically significant. * *p* < 0.05; ** *p* < 0.01. *** *p* < 0.001. Original uncropped blots of western blotting showed in Figure S7.

Next, we investigated whether iron interferes with the molecular pathways of autophagy by measuring the levels of microtubule-associated protein 1 light chain 3 (LC3) variants, LC3-I and LC3-II in representative cell lines. We treated MM.1S and U266 cells with titrated concentrations of FeAC for 24 or 72 h, in the presence or absence of bafilomycin that blocks autophagosome fusion with lysosomes. To evaluate autophagosome formation, we measured protein levels and LC3-II/LC3-I ratio in bafilomycin treated cells. To quantify autophagosome-lysosome fusion rate (autophagic flux), we considered lysosome LC3-II consumption by measuring the difference of LC3-II levels between bafilomycin treated and untreated cells. Untreated cells showed low LC3-II levels that were strongly increased in bafilomycin treated cells, suggesting high rate of LC3-II consumption and autophagic flux (Figure 3a,b). Treatment with iron for 24 h did not influence LC3 levels, nor LC3-II consumption (not shown). After 72 h, iron promoted a dose-dependent increase of both LC3-I and LC3-II proteins, increase of LC3-II/I ratio and of LC3-II consumption by lysosomes in MM.1S (Figure 3a) but not in U266 cells (Figure 3b). We concluded that prolonged iron exposure increases autophagosome formation and autophagic flux in MM.1S likely as a consequence of poly-Ub proteins accumulation.

Since autophagy upregulation is a compensatory mechanism that contrasts bortezomib toxicity [16,17], we asked whether stimulation of autophagy by iron might occur upon combination with bortezomib, partially buffering iron toxicity. We analyzed LC3 levels in U266 cells treated with 600 µM iron or 10 nM bortezomib or combination for 48 h in presence or absence of bafilomycin. As expected, addition of bafilomycin that blocks autophagy increased bortezomib and bortezomib-iron toxicity (Figure S3). As for LC3 dynamics, bortezomib strongly increased LC3-I levels while LC3-II/LC3-I ratio, LC3-II levels, and LC3-II consumption were reduced compared to bortezomib untreated cells (Figure 3c). This reduction likely occurred since in bortezomib treated cells the increase of LC3-II levels and autophagic flux was detectable in insoluble fractions (Figure 3c). Importantly, we did not appreciate variations between bortezomib and bortezomib-iron treated cells (Figure 3c), excluding that iron stimulates autophagy more than bortezomib alone. We came to the same conclusion by considering p62 levels as another autophagy marker. It deeply accumulated in insoluble fractions upon bortezomib without difference between bortezomib and bortezomib-iron treated cells (Figure 3c).

### 2.2. Iron Increases Carfilzomib Efficacy in MM Cells

**Figure 4:**
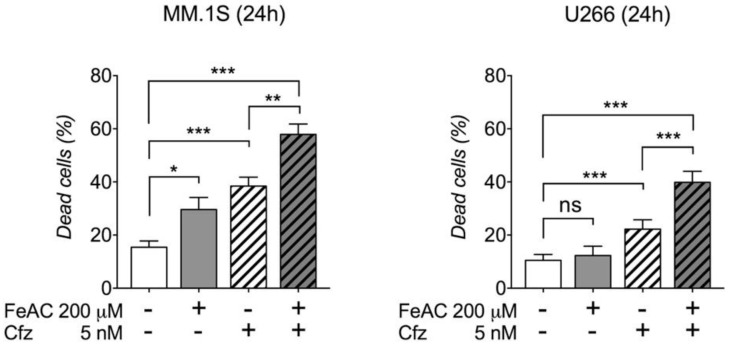
Iron increases carfilzomib efficacy in MM cell lines. Percentage of dead cells measured in MM.1S and U266 cell lines subjected to 200 μM ferric ammonium citrate (FeAC) or 5 nM carfilzomib (Cfz) or combination for 24 h. Values are shown as mean ± standard errors of at least 4 independent experiments for each cell line. Statistical differences were determined by Tukey post-ANOVA test. ns: non-statistically significant. * *p* < 0.05; ** *p* < 0.01. *** *p* < 0.001.

Motivated by translational purpose, we then asked whether iron toxicity increases the efficacy of carfilzomib, a second-generation proteasome inhibitor recently approved for MM treatment. We treated MM.1S and U266 cells with 200 µM FeAC or 5 nM carfilzomib or FeAC plus carfilzomib for 24 h. We reduced iron concentration compared to the experiments described above since here we were mainly interested in verifying the effect of carfilzomib-iron combination rather than studying the effects of iron toxicity. As expected, this iron concentration alone was not sufficient to induce cell death in U266 cells, and carfilzomib caused cell death in both cell lines according to their predicted sensitivity (Figure 4). Nevertheless, the combination caused more cell death than individual treatments, confirming the general relevance of the combination of iron toxicity with proteasome inhibition irrespective of the drug utilized (Figure 4).

### 2.3. Iron Increases the Efficacy of Bortezomib-Melphalan-Prednisone Regimen in Vk*MYC Mice

**Figure 5:**
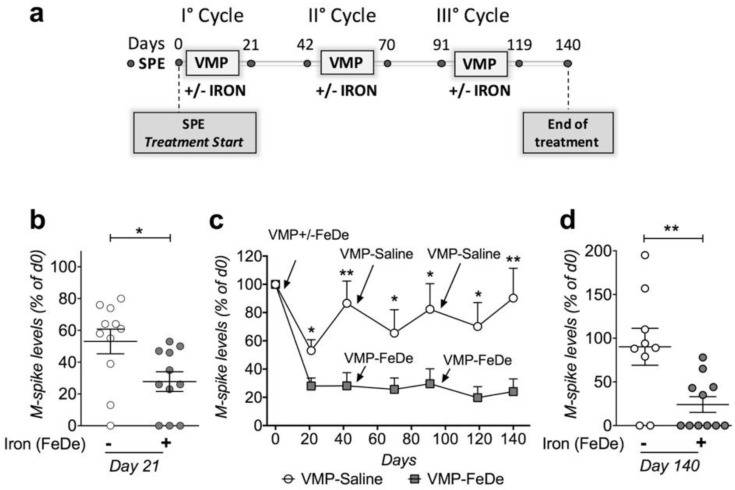
Iron improves bortezomib-melphalan-prednisone (VMP) regimen efficacy in Vk*MYC mice. (**a**) Treatment schedule. Mice were treated with VMP regimen plus/minus 100 mg/Kg iron dextran (FeDe) for 3 consecutive cycles administered at 7 weeks intervals. Disease expansion was determined by measuring serum monoclonal component (M-spike) by serum protein electrophoresis (SPE) at indicated time points. (**b**) Variation of M-spike levels from treatment start (Day 0) in VMP-Saline (n.11) and VMP-FeDe (n.11) treated mice at day 21 of the first cycle. Each circle in the scatter graph indicates M-spike reduction of each mouse analyzed. (**c**) Variation of M-spike levels from treatment start at indicated time points during follow-up. Data are shown as means +/- standard errors of M-spike variation in the 2 treatment groups. (**d**) M-spike variation at the end of treatment (day 140). Each circle in the scatter graph indicates M-spike reduction of each mouse analyzed. Two VMP-Saline mice died during the experiment. Statistically significant differences between VMP-FeDe and VMP-Saline mice were determined by t-test. * *p* < 0.05; ** *p* <0.01.

We previously showed that the bortezomib-iron combination reduces the disease more efficiently than bortezomib as a single agent in MM Vk*MYC mouse model [9]. Here, we further explored the potential therapeutic value of this observation by testing whether iron addition increases the efficacy of the VMP regimen. We treated VK*MYC mice (Monoclonal component >7%, mean age 85 ± 14 weeks) with 3 consecutive VMP-Saline or VMP-Iron cycles, according to human schedule, using drugs at human equivalent doses and iron dextran (FeDe) as iron source (Conversion of Animal Doses to Human Equivalent Doses, www.fda.gov/media/72309/download) (Figure 5a). Mice treated with VMP plus FeDe showed a greater reduction of serum monoclonal component than mice treated with VMP-Saline after the first cycle (Figure 5b). Based on monoclonal component variation, VMP-Saline mice relapsed during the following three weeks, while VMP-FeDe mice showed prolonged remission (Figure 5c). Subsequent VMP-FeDe cycles successfully controlled the disease expansion, whereas VMP-Saline treated mice progressively relapsed, becoming therapy-resistant (Figure 5c). At the end of 3 consecutive cycles, VMP-FeDe treated mice showed undetectable monoclonal component (Very good response) in 6 out of 11 cases and no case of increased monoclonal component. Differently, 2 VMP-Saline mice died without experiencing body weight loss before the end of the treatments and outlived mice showed undetectable monoclonal component in only 2 out of 9 cases and no response or progressive disease (PD) in the remaining. At sacrifice, we evaluated BM PCs expansion by flow cytometry in representative cases from opposite response categories, undetectable monoclonal component and PD. In the former mice, PCs were almost undetectable, as in wild-type normal mice, whereas PD mice showed abundant PCs, confirming that monoclonal component levels reflect BM disease expansion in these VK*MYC cases (Figure S4). We concluded that iron addition is a valuable strategy to increase VMP regimen efficacy.

Since extra iron accumulates in the liver (Figure S5), we measured serum levels of albumin, cholinesterase and alanine aminotransferase (ALT) as indexes of liver function and hepatocyte necrosis after 2 cycles and at the end of treatment. All markers remained within reference ranges during the study in both VMP-Saline and VMP-FeDe treated mice. Similarly, creatinine, a marker of kidney injury, was unchanged in all conditions (Table 1). We concluded that high-dose parenteral iron did not induce systemic toxicity in VMP-Iron treated mice.

To improve the translational relevance of our findings, in a second set of in vivo experiments we replaced iron dextran with ferric carboxymaltose (FeCM), a clinically used iron preparation, at lower dosage (20 mg/Kg). Although mice of the latter set of experiments displayed a better response to VMP than mice described above, making challenging the evaluation of the effect of iron by monoclonal component variation, we observed a trend toward monoclonal component reduction in VMP-FeCM (*p* = 0.06 vs VMP-Saline treated mice) (Figure S6A). Considering classes of responses at the end of the first cycle, VMP-FeCM treated mice showed undetectable monoclonal component in 7 out of 15 cases whereas VMP-Saline treated mice showed undetectable monoclonal component in only 1 out of 12 cases (3 mice died before the first check-point on day 21) (Figure S6A). Toxicity of VMP treatment became relevant during the follow-up. Eight VMP-Saline mice died without experiencing body weight loss before the end of the second cycle, for a total of 11 out of 15 mice, making impossible any comparison with VMP-FeCM mice (Figure S6B). As for the latter, no mouse died during treatment, and the second VMP-FeCM cycle successfully controlled the disease (Figure S6B). We concluded that Vk*MYC mice benefit of FeCM addition in terms of both tolerance and response to VMP.

## 3. Discussion

Based on the finding that MM cells are sensitive to iron toxicity, here we demonstrate that excess iron has multi-target effects in MM cells and propose that high-dose iron administration can be exploited for combination therapy to improve the disease control. We investigated the molecular pathways affected by iron toxicity and whether they interfere with the bortezomib function in four MM cell lines. Among them, MM.1S and U266 cells are commonly used as models of relatively bortezomib-sensitive and -resistant MM cells, respectively. We previously showed, and confirm here, that the latter cells show a similar range of sensitivity when exposed to iron excess [9], suggesting possible interconnection between the effects induced by iron and proteasome inhibitors.

### 3.1. Molecular Mechanisms of Iron Toxicity in MM Cells

Toxicity of iron overload is predicted to occur because of iron pro-oxidative properties since excess iron may react with hydrogen peroxide through the Fenton reaction, generating ROS. Plasma cells are intrinsically sensitive to iron toxicity because of the continuous production of high levels of hydrogen peroxide, a byproduct of Ig synthesis and secretion [19,20,21,22]. Although malignant transformation is accompanied by increased iron tolerance for proliferation [1,23], MM cells remain more sensitive to iron toxicity than other cell types [9]. 

A number of issues should be considered to explain the entity of iron-induced damage. First, iron sensitivity depends on ferritin levels, since ferritin safely stores iron, buffering its pro-oxidant activity [24]. Thus, MM cells with high basal ferritin levels, such as U266, appear equipped to better counteract iron excess than cells with low basal ferritin levels, as MM.1S [9,10]. Second, iron sensitivity depends on the efficacy of cell response to iron. Bortezomib interferes with this physiological response by blocking ferritin increase and TFR1 reduction, maximizing iron toxicity [10]. Finally, the severity of damage ultimately depends on iron concentration. Thus, treatment with 200 µM FeAC causes cell death in MM.1S but not in U266 (Figure 4), while 600 µM affects both cell lines (Figure 1a).

Iron exposure increases MDA formation, the final product of polyunsaturated fatty acids (PUFA) peroxidation and fragmentation. PUFA are highly oxidation-sensitive molecules, and their peroxidation occurs at a low level in basal growth conditions. This event is physiologically counteracted by glutathione peroxidase 4 (GPX4), which catalyzes the GSH-dependent reduction of lipid peroxides [25,26]. When the rate of PUFA peroxidation overcomes cell buffering capacity, cells undergo death by ferroptosis [25]. Iron is indispensable for this pathway since it catalyzes the formation of soluble lipid radicals that can initiate and propagate PUFA oxidation and fragmentation. Accordingly, iron chelation prevents ferroptotic cell death [27]. We found that iron exposure causes the opposite, overcoming antioxidant machinery, and leading to cell death. As documented in U266 cells, MDA formation occurs early after iron exposure and attenuates with time, probably secondary to ferritin upregulation. Indeed, combination with bortezomib that blocks ferritin increase prolongs and intensifies MDA production, exacerbating cell death.

Adaptation to high-rate lipid peroxidation has an important role in cancer development. Cancer cells become strongly dependent on GPX4 and on the cystine/glutamate antiporter (system Xc-) to import extracellular cysteine, essential for GSH biosynthesis [28,29]. Thus, the development of compounds inducing ferroptosis is emerging as a novel anticancer therapeutic strategy. Erastin, a system Xc- inhibitor, improves the efficacy of chemotherapy in several cancer cells [30,31,32]. So far, only inhibition of the antioxidant system has been explored as a strategy to induce ferroptosis. Here, we show that ferroptosis can be also induced by altering iron homeostasis, the second arm of lipid peroxidation rate control, a strategy worth to be explored even beyond MM.

A key finding in MM models is that iron toxicity increases the efficacy of bortezomib, suggesting that combination with iron supplementation might represent a potential novel strategy to overcome resistance to proteasome inhibitors. In MM.1S cells, high bortezomib sensitivity has been associated with low proteasome capacity and constitutive high proteasome workload, while the opposite occurs in U266, characterized by relatively high proteasome capacity and low workload [15]. We previously showed that bortezomib, as iron sensitivity, is inversely related to basal ferritin content, since high ferritin levels, by reducing the free iron pool, probably attenuate the bortezomib pro-oxidant effects secondary to proteasome inhibition [10]. Importantly, although iron and bortezomib sensitivity goes in parallel, bortezomib-iron combination induced death of U266 and OPM-2, which are poorly sensitive to individual treatments. Iron also potentiates the effect of carfilzomib, a second-generation drug recently approved for MM treatment, suggesting that the beneficial effect of iron supplementation might be generally applied to proteasome inhibitors-based treatments. 

The finding that iron excess impairs proteasome activity reveals that mechanisms of iron toxicity might not be limited to alteration of oxidative homeostasis. Ferrous iron impairs proteasomal chymotrypsin-like activity of purified 26S particle and of MM cellular extracts in a dose-dependent manner in vitro. The reversible inhibition of proteasome activity suggests that iron likely acts by steric interference rather than by causing oxidative modifications. Iron impairs proteasome functionality also in iron-exposed cells, as documented by iron-dependent accumulation of poly-Ub proteins. This event occurs in highly iron sensitive cells, such as MM.1S and H929, likely facilitated by an unfavorable load versus capacity ratio, as previously documented in MM.1S cells [15]. Differently, poly-Ub accumulation does not occur in the relatively low iron sensitive U266 and OPM-2, in which the inhibitory effect of iron on proteasome activity is likely buffered by a more favorable load versus capacity balance [15]. These data suggest that proteasome impairment is a determinant of iron toxicity, and that proteasome levels contribute to define iron sensitivity. More importantly, the results indicate that reduction of proteasome activity by iron cumulates that induced by bortezomib since poly-Ub protein accumulation is greater than that induced by bortezomib as a single agent. The iron efficacy occurs in combination with different proteasome inhibitors, as documented by experiments with MG132. In this context, iron might be able to overcome the increased proteasome capacity, which occurs through increased transcription and translation of proteasome subunits, a hallmark of bortezomib resistance [33].

As for the effect of iron on autophagy, the results obtained in MM cell lines exposed to increasing iron doses suggest that iron does not directly influence autophagosome formation or autophagy flux. Notwithstanding, autophagy stimulation might occur after iron exposure as a compensatory mechanism of poly-Ub proteins accumulation, as it occurs in low proteasome capacity cells, such as MM.1S. This event might limit iron toxicity when iron is combined with proteasome inhibitors since autophagy-increase promoted by iron might potentiate that promoted by bortezomib, which is a recognized mechanism of drug-resistance [16,17]. As expected, bortezomib increases autophagy. Still, iron has limited, if any, effect on this process.

### 3.2. Iron Is a Potential Candidate for MM Combination Therapy

In this work, we demonstrate the value of exploiting iron toxicity in combination with a common clinical protocol of MM therapy. This issue was not demonstrated in our previous work that exclusively tested the proteasome inhibitor in combination with high-dose iron [9]. In view of translational applicability, here we also tested both the reduction of FeDe dose used in previous studies, and multiple iron administrations. We added iron to the VMP regimen, a first-line therapy for newly diagnosed patients, especially for those considered non-fit for intensive treatment and autologous stem cell transplantation, as the elderly [34]. Iron increased VMP response and prolonged remission without causing evident systemic toxicity, as documented by normal liver and kidney function tests.

To underscore the clinical relevance of our approach, it should be noted that VMP is typically adjusted and/or dose-reduced based on patient age, disease stage, and comorbidities. The intensity reduction often leads to suboptimal and incomplete responses that, according to our data, could be improved by iron, without adding systemic toxicity. The dose of bortezomib we used (0.25 mg/Kg) was equivalent to the human dose (0.75 mg/m^2^) administered to old and frail patients. Similarly, iron dextran dose used in mice (100 mg/Kg), once converted to human dose, is within the range of iron dosages (up to 20 mg/Kg) that may be used to treat iron-deficient patients in a single injection. Since iron dextran, commonly used in preclinical studies, is rarely employed in clinical practice because of potentially severe allergic reactions [35], we moved to FeCM as an alternative iron source that we used in mice at 20 mg/Kg, the maximal iron dose advised in humans. This iron preparation is largely used without evident toxicity in both absolute and functional iron deficiency [36,37,38], the latter condition being common in MM patients. 

Our results revealed the potential toxicity of VMP regimen for frail subjects, as denoted by high mortality in old Vk*MYC mice before the end of programmed treatment in the VMP-Saline arms. Irrespective of the specific sensitivity showed by the 2 different pools of mice, iron administration increased VMP efficacy and fully prevented these adverse events in both the experiments. Considering the need of balancing treatment efficacy with limiting side effects, overall our pre-clinical data validate iron as a candidate for combination therapy with VMP.

## 4. Materials and Methods

### 4.1. Cell Culture

Human MM cell lines MM.1S, U266 and H929 were purchased from ATCC, OPM-2 cells were from DSMZ-German Collection of Microorganisms and Cell Cultures. All cells were cultured in RPMI-1640 medium (Euroclone, Pero, Italy) supplemented with 10% fetal bovine serum (Thermo-Scientific, Waltham, MA, USA), 2 mM L-glutamine, 100 U/mL penicillin and 100mg/mL streptomycin (Sigma–Aldrich, St. Louis, MO, USA). Cells (1.5 × 10^6^) were seeded at 5 × 10^5^ cells/mL and grown in presence or absence of 10 nM bortezomib (LC Laboratories, Woburn, MA, USA,) and/or 600 µM ferric ammonium citrate (FeAC) (Sigma–Aldrich). Alternatively, bortezomib was replaced by 5 nM carfilzomib (Amgen, Thousand Oaks, CA, USA). At indicated times, cells were collected, diluted 1:1 with trypan blue exclusion dye (Bio-Rad, Hercules, CA, USA) and counted using TC20™ Automated Cell Counter (Bio-Rad). Cell pellets were then processed for malondialdehyde determination or western blotting. In a second experimental setting, cells were grown in presence of titrated doses of FeAC (100, 300 and 600 μM), and cell pellets processed for western blotting.

### 4.2. Lipid Peroxidation

Lipid peroxidation was determined using the Lipid Peroxidation Assay Kit (Sigma–Aldrich) following manufacturer instructions. Malondialdehyde levels were quantified using the Victor 3 Multilabel Counter (Wallac, Perkin Elmer, Waltham, MA, USA) at 532 nm for excitation and 553 nm for emission. Finally, results were normalized on cell count.

### 4.3. 26S peptidase Activity Measurements

Chymotryptic activity of purified 26S rabbit proteasome [39] was measured using the specific fluorogenic substrate Suc-LLVY-amc (Bachem, Bubendorf, Switzerland) as already described [40]. Proteasomal chymotrypsin-like activity was assessed in MM cell extracts according to published methods [41]. Briefly, cells were centrifuged and pellets incubated in ice-cold extraction buffer (50 mM Tris/HCl, pH 7.5, 1 mM DTT, 0.25 M sucrose, 5 mM MgCl_2_, 0.5 mM EDTA, 2 mM ATP, 0.025% digitonin) for 5 min and extracts prepared by centrifugation for at 20.000 *g* for 30 min at 4 °C. The inhibitory effects of iron were studied by pre-incubating 26S or cellular extracts with ferrous chloride or ferrous sulfate for 5 min and by comparing the residual activity with that of corresponding control anions used at the same molar concentration. Suc-LLVY-AMC was used at a final concentration of 100 µM in 20 mM Tris-HCl, pH 7.5, 1mM ATP, 2 mM MgCl_2_, and 0.2% BSA. Reactions were started by adding an aliquot of 26S or cellular extract, and the fluorescence of released AMC (excitation, 380 nm; emission, 460 nm) was monitored continuously at 37 °C with a Cary Eclipse spectrofluorometer (Varian, Santa Clara, CA, USA). Assays were calibrated using standard solutions of the free fluorophore, and the reaction velocities were calculated from the slopes of the initial linear portions of the curves. Substrate consumption at the end of incubation never exceeded 1%. In cellular extracts, background activity (caused by non-proteasomal degradation) was determined by addition of 2 µM epoxomicin (Enzo Life Science, NY, USA) and subtracted from total chymotrypsin-like activity [42].

### 4.4. Mouse Studies

Mice were bred in the pathogen-free animal facility of the IRCCS Ospedale San Raffaele in accordance with the European Union guidelines. The study was approved by the local Institutional Animal Care and Use Committee and by the Italian Ministry of Health (225/2015-PR). Vk*MYC mice (n: 22; monoclonal component > 7%) were treated with 0.25 mg/Kg bortezomib (i.p.) (Millennium Pharmaceuticals, Cambridge, MA, USA) at days 1,4,8,11 plus 2 mg/Kg melphalan (i.p.) (Denza Join Enterprise, Malaysia) and 20 mg/Kg prednisone (p.o.) (Caesar & Loretz GmbH, Hilden, Germany) at days 1–4 (VMP cycle). We replicated VMP cycle after 7 weeks for a total of 3 times. A group of mice (*n* = 11) was additionally treated with 100 mg/kg iron dextran (i.p.) (Sigma–Aldrich) at day 5 of every cycle. We followed treatment response by measuring monoclonal component (M-spike) levels by serum protein electrophoresis at days 21, 42, 70, 91, 119, 140 after treatment start. A second pool of Vk*MYC mice (n: 30; monoclonal component > 7%) was treated with VMP as describe above, for 2 cycles. A group of these mice (*n* = 15) was additionally treated with 20 mg/kg of ferric carboxymaltose (i.v.) (Vifor Pharma, St. Gallen, Switzerland) at day 5 of every cycle. M-spike levels were measured at days 21, 42, 70, 91 after treatment start. Within each experimental setting, treatment groups were matched for weight, age and disease level.

## 5. Conclusions

We report that iron exposure has multiple toxicities in MM cells, ultimately causing their death. Iron generates oxidative stress and lipid damage and inhibits proteasome functionality, thus interfering both with redox and protein homeostasis, essential processes for MM cell survival. These pre-clinical results pave the way to design pilot clinical trials that include iron administration to increase the efficacy of current MM therapies, such as VMP regimen. This approach might be tested in elderly frail patients affected by multiple comorbidities who often have incomplete responses to VMP dose reduction, and whose treatment still remains a major therapeutic challenge.

## Figures and Tables

**Table 1:** Serum indicators of liver and kidney damage and functionality.

Peripheral Blood Markers	Treatment Start		After Two Cycles		End of Treatments		References Values
	VMP−Saline	VMP−FeDe	VMP−Saline	VMP−FeDe	VMP−Saline	VMP−FeDe	
Albumin (g/dl)	3.1 ± 0.35	3.1 ± 0.46	3.1 ± 0.26	3.3 ± 0.26	3.0 ± 0.42	2.7 ± 0.36	2.7–3.6
Cholinesterase (U/L)	4490 ± 1001	5343 ± 1623	5449 ± 305	5956 ± 1495	4939 ± 297	4998 ± 1376	1400–6300
Alanine aminotransferase (U/L)	51 ± 14	41 ± 13	45 ± 11	46 ± 14	31 ± 6	55 ± 9	<70
Creatinine (mg/dL)	−	−	−	−	0.31 ± 0.03	0.29 ± 0.02	0.31–0.40

## Supplementary Materials

The following are available online at https://www.mdpi.com/2072-6694/12/4/970/s1, Figure S1: Iron triggers cell death by inducing lipid damage in H929 and OPM-2 cells, Figure S2: Iron impairs proteasomal activity and causes polyubiquitinated proteins accumulation, Figure S3: Bafilomycin increases bortezomib and bortezomib-iron toxicity, Figure S4: Representative flow cytometry analysis of bone marrow samples of VK*MYC mice, Figure S5: Liver iron content of Vk*MYC mice, Figure S6: Ferric carboxymaltose improves VMP regimen efficacy in Vk*MYC mice, Figure S7: Original uncropped blots of western blotting showed in Figure 3. Supplementary Methods: Western blotting; Serum protein electrophoresis; Liver and kidney functionality; Flow cytometry of bone marrow samples; Analysis of liver iron content; Statistical analyses.
