# Supplementary material for: Iron Causes Lipid Oxidation and Inhibits Proteasome Function in Multiple Myeloma Cells: A Proof of Concept for Novel Combination Therapies
Source: Cancers (Basel). 2020 Apr 14;12(4):970. doi: 10.3390/cancers12040970 (PMC7226326; doi:10.3390/cancers12040970)

Supplementary material

Supplementary Figure 1

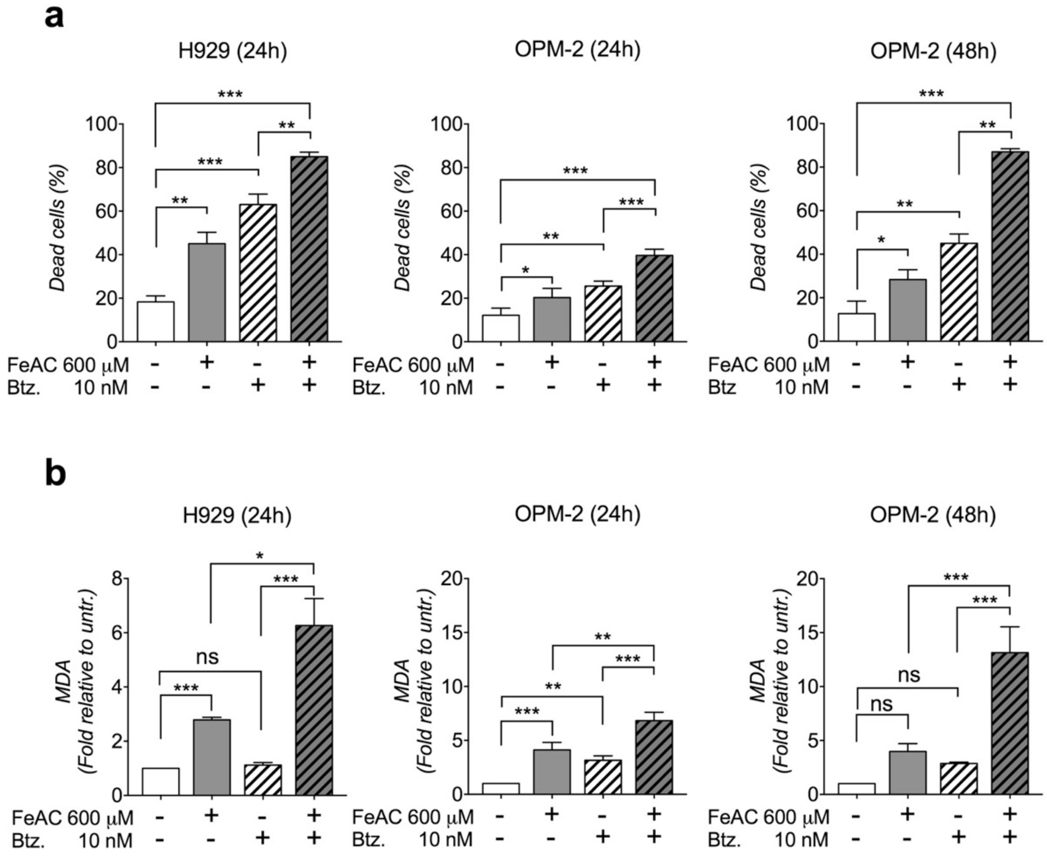

Figure S1. **Iron triggers cell death by inducing lipid damage.** H929 and OPM-2 cell lines were subjected to 600 μM ferric ammonium citrate (FeAC) or 10 nM bortezomib (Btz) or combination for 24 or 48 hours. (**a**) Percentage of dead cells. (**b**) Malondialdehyde (MDA) levels presented as fold change relative to untreated cells. Values are shown as mean +/- standard errors of at least 4 independent experiments for each cell line. Statistical differences were determined by Tukey post ANOVA test. ns: non-statistically significant. *p<0.05; **p<0.01. ***p<0.001.

Supplementary Figure 2

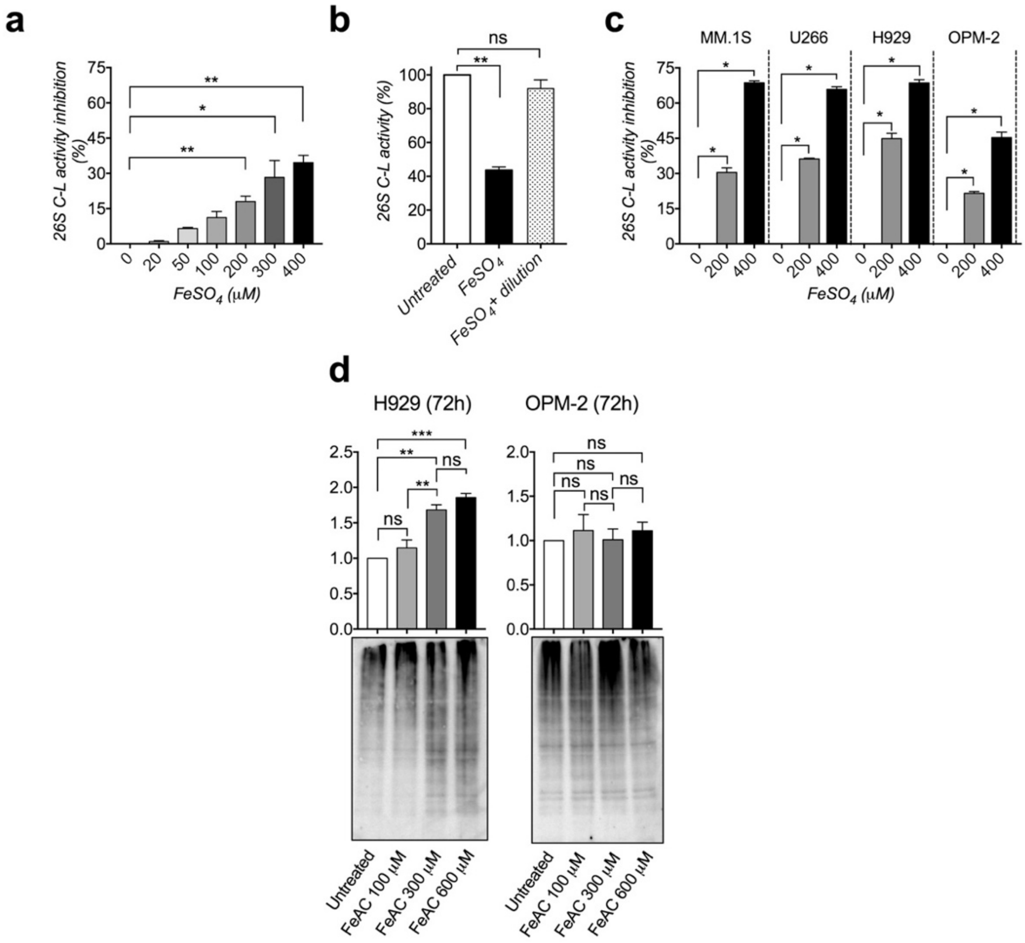

Figure S2. Iron impairs proteasomal activity and causes polyubiquitinated proteins accumulation. (**a-b**) Evaluation of chymotrypsin-like (C-L) activity of purified 26S proteasome after pre-incubation with titrated doses of ferrous sulfate ($FeSO_4$) for 5 minutes. (**a**) Data show the percentage of C-L activity inhibition. (**b**) Data show residual C-L activity after pre-incubation with 400 µM $FeSO_4$ followed or not by iron dilution prior of C-L activity evaluation. (**c**) Evaluation of proteasomal C-L activity of MM cellular extracts after pre-incubation with titrated doses of $FeSO_4$ for 5 minutes. Background activity (caused by non-proteasomal degradation) was determined by addition of 2 µM epoxomicin and subtracted from total C-L activity. (d) Polyubiquitinated (Poly-Ub) protein levels in H929 and OPM-2 cells treated with titrated doses of ferric ammonium citrate (FeAC) for 72 hours. Upper panels: summary of densitometry of at least 3 independent experiments (Fold relative to untreated). Lower panels: Representative western blotting. Values are shown as mean +/- standard errors. (**a-c**) Statistical differences were determined by nonparametric Mann-Whitney U test. (**d**) Statistical differences were determined by Tukey post ANOVA test. ns: non-statistically significant. *p<0.05; **p<0.01. ***p<0.001.

Supplementary Figure 3

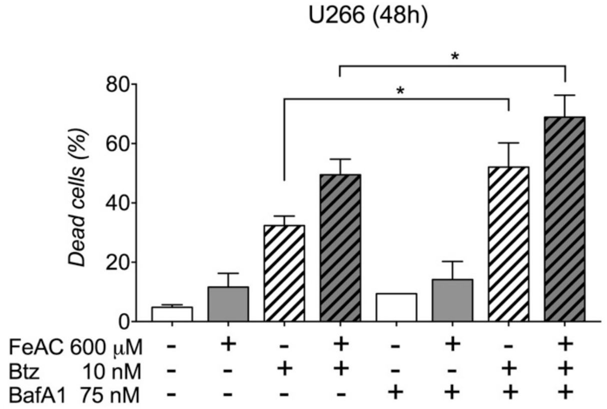

Figure S3. Bafilomycin increases bortezomib and bortezomib-iron toxicity. U266 cells were subjected to 600 µM ferric ammonium citrate (FeAC) or 10 nM bortezomib (Btz) or combination for 48 hours. Where indicated, bafilomycin (BafA1) was added for the last 8 hours of incubation. Bars indicate percentage of dead cells. Values are shown as mean +/- standard errors of 3 independent experiments. Statistical differences discussed in the text were determined by Tukey post ANOVA test. *p<0.05.

Supplementary Figure 4

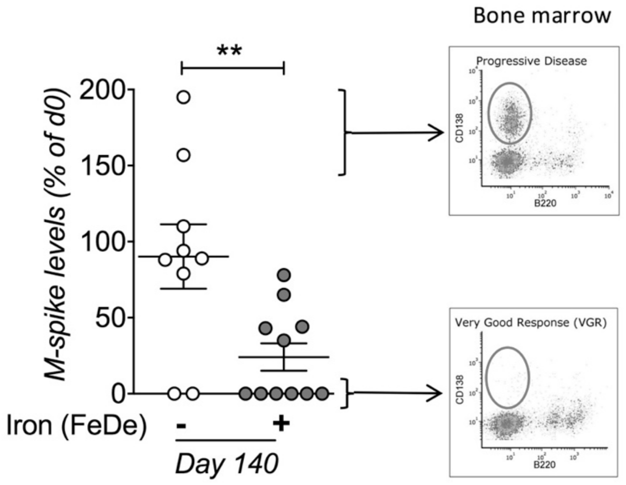

Figure S4. Left panel. Duplicate of Figure 5D, showing M-spike variation at the end (day 140) of 3 consecutive VMP cycles +/- iron dextran (FeDe), as described in Figure 5. Each circle in the scatter graph indicates M-spike reduction of each mouse analyzed. Right panels. Representative flow cytometry analysis showing $CD138^{+}B220^{-}$ cells (Multiple myeloma cells) in bone marrow samples from Vk*MYC mice with very good response or progressive disease at the end of the treatment (Day 140). Arrows indicate relationship between response categories and representative MM cells in the bone marrow.

Supplementary Figure 5

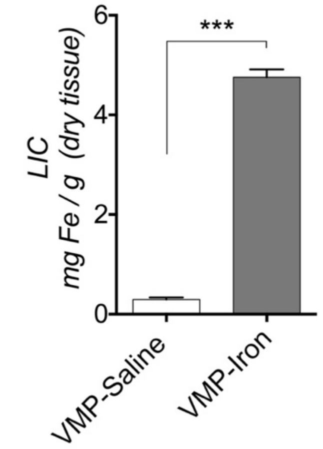

Figure S5. Liver iron content (LIC) of Vk*MYC mice described in Figure 5. LIC was measured at the end of 3 consecutive VMP-Saline or VMP-Iron (FeDe) cycles. Statistical differences were determined by t-test. ***p<0,001.

Supplementary Figure 6

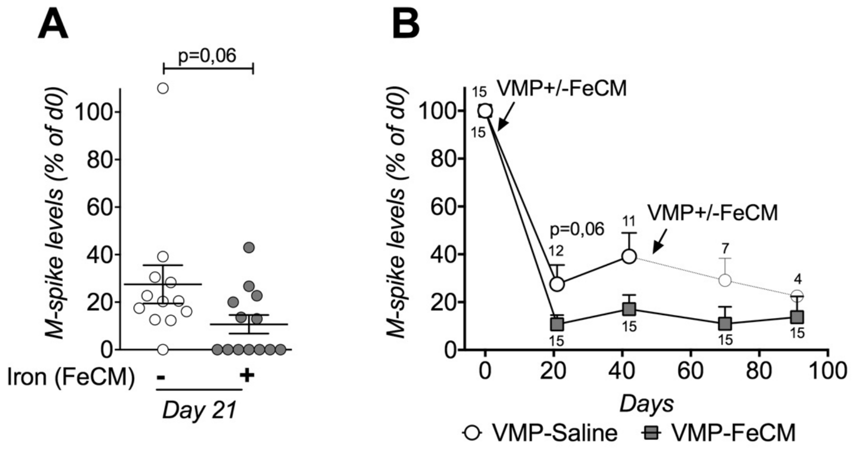

Figure S6. Ferric carboxymaltose improves VMP regimen efficacy in Vk*MYC mice. Vk*MYC mice were treated with bortezomib-melphalan-prednisone schedule (VMP) plus/minus 20 mg/Kg ferric carboxymaltose (FeDe) for 2 consecutive cycles administered at 7 weeks intervals. Disease expansion was determined by measuring serum monoclonal component (M-spike) by serum protein electrophoresis (SPE) at indicated time points. (A) Variation of M-spike levels with respect to treatment start (Day 0) in VMP-Saline (n.15) and VMP-FeCM (n.15) treated mice at day 21 after the first cycle. Each circle in the scatter graph indicates single mice analyzed. Three VMP-Saline mice died before day 21. (B) Variation of M-spike levels with respect to treatment start at indicated time points during follow up. Data are shown as means +/- standard errors of M-spike variation in the 2

treatment groups. Numbers indicate mice analyzed. 1 VMP-Saline mice died between days 21 and 40. Dashed line: Other 8 VMP-Saline mice died after the check point of day 40 and before the end of the second cycle (Day 91), for a total of 11 out of 15 mice, making impossible any comparison with VMP-FeCM mice. Statistically significant differences between VMP-FeCM and VMP-Saline was determined at day 21 by t-test.

Supplementary Methods

Western blotting

Cell pellets were lysed in RIPA buffer (100 mM Tris–HCl pH 8.0, 140 mM NaCl, 0.5 mM EDTA, 1% Deoxycholic Acid, 1% Triton X-100 and 0.1% SDS) for 15 minutes on ice. After centrifugation at 13000g for 10 min at 4°C, supernatants were collected, and protein concentration measured using BCA kit (Thermo-Fisher Scientific) according to the manufacturer protocol. Insoluble fractions obtained by centrifugation were resuspended in Laemmli sample buffer 2X.

Proteins were evaluated by western blotting after SDS-PAGE using standard techniques. Briefly, blots were blocked with 5% bovine serum albumin (BSA, Sigma-Aldrich) in TBS (0.5M Tris-HCl pH 7.4 and 0.15M NaCl) containing 0.1% Tween 20 (TBST). Then, blots were incubated overnight with rabbit anti-LC3 (1:1000 dilution; Cell signaling) or for 1h with mouse anti-Ub (1:200 dilution; Santa Cruz) or mouse anti-β-actin (1:10000 dilution; Sigma-Aldrich) primary antibodies diluted in TBST with 5% BSA followed by 1 hour incubation with secondary HRP-conjugated antibodies (1:50000 in TBST with 5% BSA) followed by 1 hour incubation with secondary HRP-conjugated goat anti-rabbit and rabbit anti-mouse, respectively (1:50000 in TBST with 5 % BSA), respectively (Sigma-Aldrich). Signals were revealed by ECL Prime (GE-Healthcare). Densitometry was performed using ImageLab BIORAD software.

Serum protein electrophoresis (SPE)

Serum protein electrophoresis (SPE) was performed on the Hydrasys instrument (Sebia, Florence, Italy). Briefly, 10 μL of sera were applied to the Hydragel agarose gels (Sebia). Electrophoresis, drying, amidoblack staining, de-staining and final drying were carried out automatically. Gels were scanned with Hydrays 2 Scan densitometer and scanning resulting profiles were analyzed by Phoresis software (Sebia).

Liver and kidney functionality

Serum indicators of liver and kidney functionality and damage were assessed by ILab Aries analyzer (Instrumentation Laboratory, Werfen Group, Milan, Italy), using an enzymatic assay for cholinesterase, alanine amino transferase (ALT) and a colorimetric method to detect creatinine and albumin. All parameters were detected using kits and controls provided by ILab Aries. Experiment precision was determined running the standard controls before each determination and the values obtained for controls were always within the expected ranges.

Flow cytometry of bone marrow samples

Bone marrow cell suspensions were filtered, centrifuged and suspended in PBS containing 0.1 % BSA. Cells were stained with PE-Cy7-conjugated anti-B220 (clone RA3-6B2; BD Bioscience) and APC-conjugated anti-CD138 (clone 281-2; BD Bioscience). After washing, cells were analyzed on Navios Flow Cytometer (Beckman Coulter). Analyses were performed with the FCS Express software (DeNovo Software).

Analysis of liver iron content

Liver (LIC) and spleen (SIC) iron contents were determined as described in (Rausa M. et al, 2015). Briefly, tissue samples were dried at 110°C for 72 hours, weighed, and digested in acid solution for

20 hours at 65°C. After digestion, 1 mL of working chromogenic reagent (1 volume of 0.1% bathophenanthroline sulfate and 1% thioglycolic acid solution, 5 volumes of water, and 5 volumes of saturated sodium acetate) was added to 20 µl of acid extract and the solutions were incubated for 30 minutes at room temperature. Finally, samples were red at 535 nm and absorbance compared with iron standard curve.

Statistical analyses

Statistically significant differences were determined as indicated in figure legends. T-test and Tukey post ANOVA test were performed using GraphPad Prism Version 7.0a. To compare chymotrypsin-like activities we adopted a nonparametric Mann-Whitney U test. Software SPSS.

Supplementary Figure 7. Original uncropped blots of western blotting showed in Figure 3

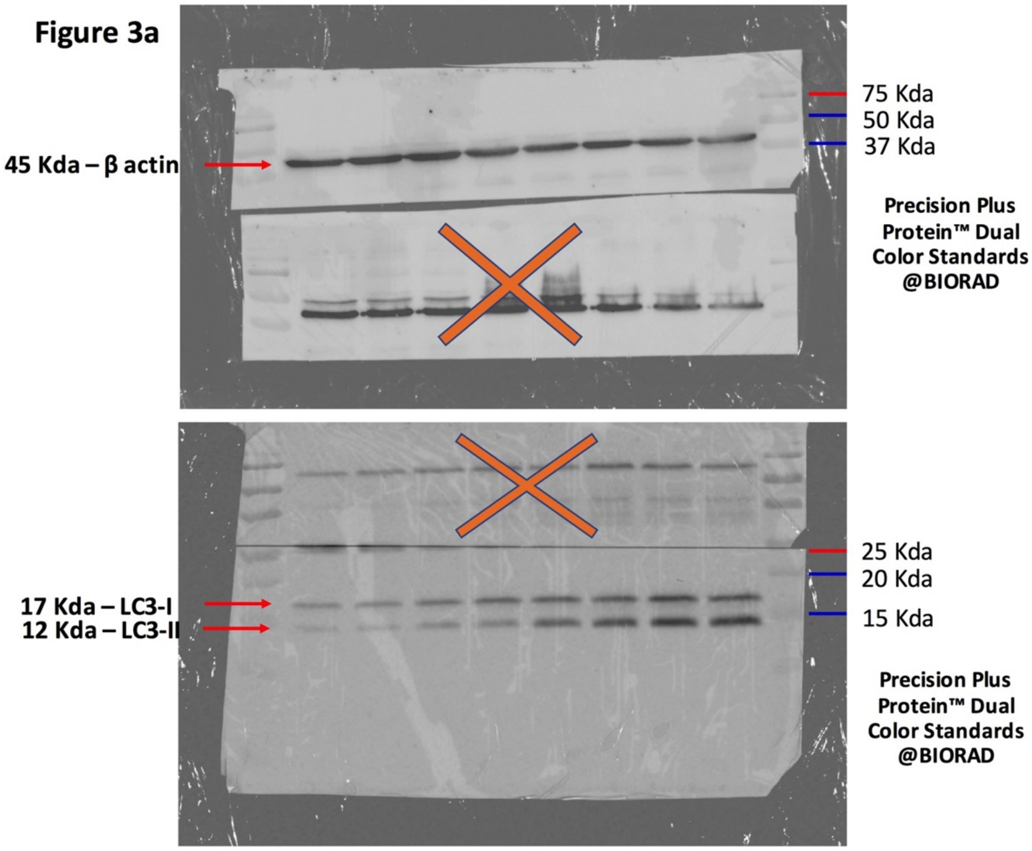

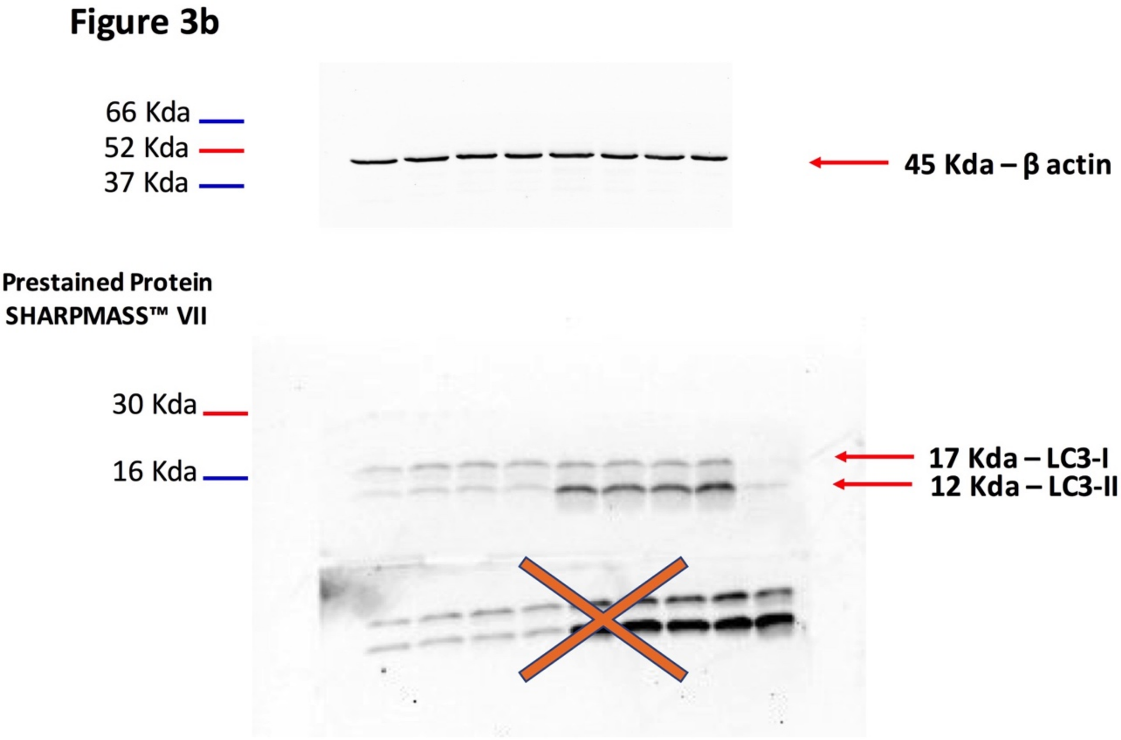

Figure 3c

(soluble)

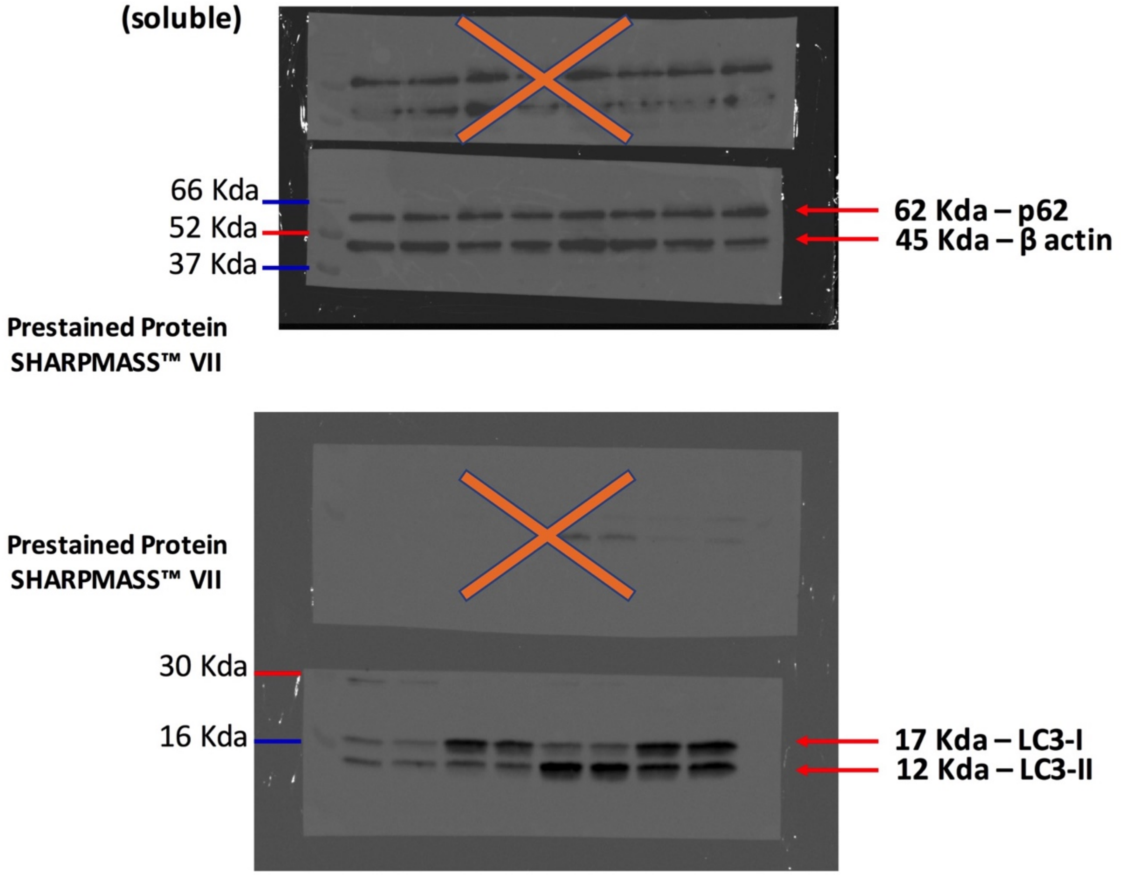

Figure 3c

(insoluble)

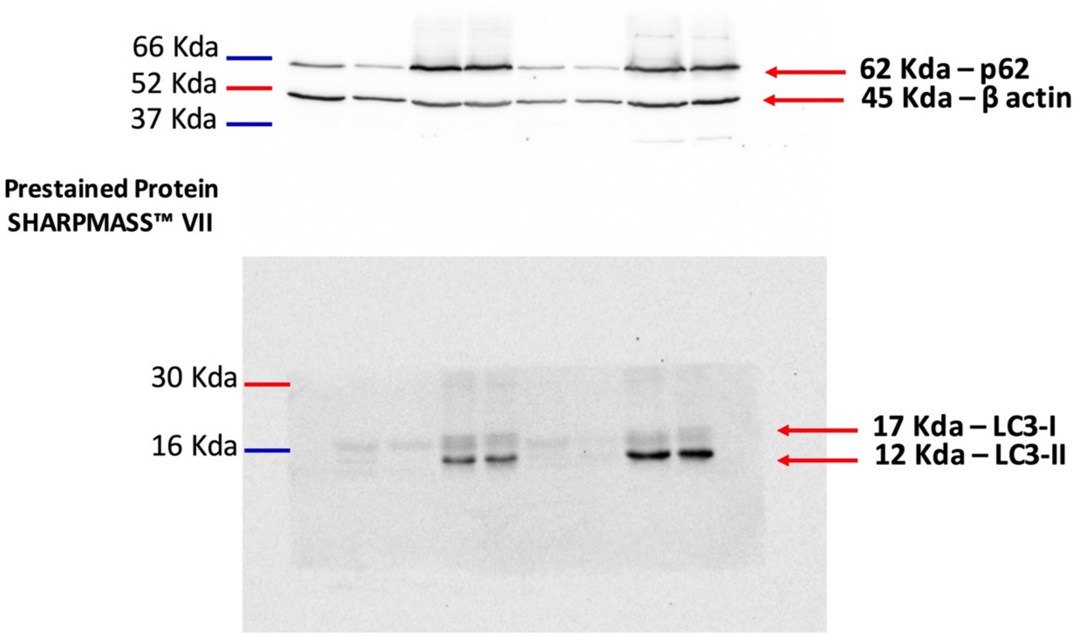

Supplement: Supplementary file 1 [file cancers-12-00970-s001.pdf]
